# Une cause exceptionnelle des péritonites: une perforation iléale par un corps étranger lors de la réduction d’une hernie inguinale

**DOI:** 10.11604/pamj.2018.31.37.11758

**Published:** 2018-09-18

**Authors:** Mohamed Moez Kammoun, Rim Karray, Kais Regaieg, Mabrouk Bahloul, Mounir Bouaziz

**Affiliations:** 1 Service de Réanimation Médicale CHU Habib Bourguiba Route El Ain, Sfax, Tunisie

**Keywords:** Péritonite secondaire, hernie, corps étranger, réanimation, choc septique, Secondary peritonitis, hernia, foreign body, resuscitation, septic shock

## Abstract

La péritonite secondaire est fréquemment rapportée dans la littérature. Les causes sont multiples. Nous rapportons un cas d'une perforation iléale par une cause exceptionnelle. Il s'agit d'une perforation iléale par un corps étranger lors de la réduction d'une hernie inguinale, responsable d'une péritonite grave avec tableau de défaillance multiviscérale.

## Abstract (en)

Secondary peritonitis is frequently reported in the literature. It is due to multiple causes. We report an exceptional cause of ileal perforation due to a foreign body during reduction of an inguinal hernia. It caused severe peritonitis with multiple organ failure.

## Introduction

La péritonite aiguë est une inflammation du péritoine qui se développe suite à une contamination du péritoine par des micro-organismes, des composés chimiques irritants ou les deux [1]. Parmi les péritonites d'origine infectieuse, on distingue les péritonites primitives (ou encore primaires, spontanées ou idiopathiques) des péritonites secondaires lorsqu'il existe un foyer intra-abdominal responsable de l'infection. Cette dernière peut être secondaire à une performation viscérale, un processus inflammatoire survenant dans la cavité abdominale ou à son voisinage, ou encore secondaire à une plaie pénétrante [1]. Parmi les causes des perforations digestives, la réduction d'une hernie inguinale a été rapportée depuis 1939 [1]. Cependant, une péritonite secondaire à une perforation iléale par un corps étranger lors de la réduction d'une hernie inguinale n'a nos connaissances jamais été rapportée. Nous rapportons un cas une péritonite secondaire à une perforation iléale par un cure dent lors de la réduction d'une hernie inguinale.

## Patient et observation

**Figure 1:**
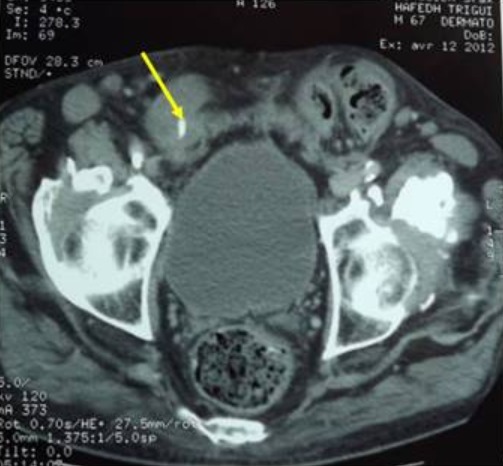
Le scanner abdominal montrant le corps étranger (indiqué par la flèche) dans la lumière intestinale

Il s'agit d'un patient âgé de 67 ans aux antécédents d'hypertension artérielle équilibré sous traitement, d'érysipèle du membre inférieur droit hospitalisé en réanimation pour réanimation post opératoire d'une péritonite secondaire. Le patient a été hospitalisé le 21 Mars 2012 au service de dermatologie pour érysipèle de la jambe droite. Lors de son séjour, il a présenté une douleur abdominale avec vomissement sans arrêt des matières et des gaz. L'examen clinique a montré un abdomen souple dépressible, pas de notion d'arrêt des matières et des gaz avec présence d'une hernie inguino-scrotale engouée. Cette hernie a été réduite avec succès par les chirurgiens. Quarante huit heures après la réduction de l'hernie, le patient a développé une douleur abdominale diffuse avec distension abdominale et arrêt des matières et des gaz. L'examen clinique trouve un patient fébrile à 39°C, un état hémodynamique stable (PA: 120/60 mmHg, Pouls à 100/min) et une contracture abdominale généralisée. Le bilan biologique a montré une hyperleucocytose à 12250 élts/mm^3^, un taux d'hémoglobine à 12,6 g/dl, un bilan d'hémostase correct (plaquettes : 295000 élts/mm^3^; TP: 73% et TCA: 30/30) et un bilan rénal normal (urée: 10 mmol/l et créatininémie à 72 µmol/l). Un scanner abdominal a été réalisé en urgence et a montré un aspect en faveur d'une occlusion intestinale aigue grélique en amont d'un niveau transitionnel grélique distal en regard d'une hernie inguinale droite. Présence d'une péritonite localisée au niveau de la fosse iliaque droite. Une hernie inguinale droite siège d'une anse grélique comportant un corps étranger (Figure 1). Présence d'un épanchement intra-péritonéal de moyenne abondance.

**Figure 2:**
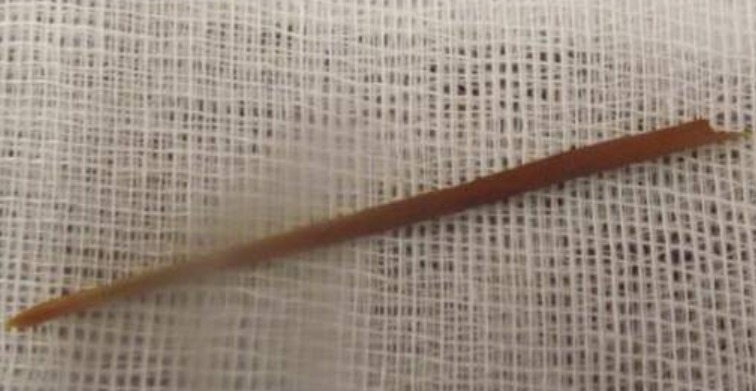
Le corps étranger après son ablation

Le diagnostic d'une péritonite aigu a été retenu et le patient a été opéré en urgence. En per opératoire on découvre un épanchement purulent intra-péritonéal de grande abondance avec présence de fausses membranes et des anses grêliques très dilatées. L'exploration du grêle a permis de trouver un corps étranger (cure dent) sans perforation décelable. L'épreuve aux bleu de méthylène a révélé une perforation grélique (iléale à 20 cm du carrefour iléo-coecal). Un prélèvement bactériologique a été fait. De plus, une toilette péritonéale, une stomie sur baguette avec cure de l'hernie ont été réalisées, le corps étranger a été enlevé (Figure 2) et le patient a été admis en réanimation pour complément de prise en charge. A l'admission en réanimation, le patient a développé un état de choc septique nécessitant en plus des antibiotiques (Imipinème, Amiklin et flagyl) du remplissage vasculaire le recours aux catécholamines (Noradrénaline à la dose de 5mg/heure). Les prélèvements microbiologiques pratiqués en per-opératoire ainsi que les hémocultures pratiquées à l'admission ont poussé à enterobacter sakazokii et Escherichia coli sensibles à l'antibiothérapie prescrite. Durant son séjour en réanimation, le patient a développé un tableau de défaillance multiviscérale avec une CIVD, une insuffisance rénale aigue (urée à 15 mmol/l, une créatininémie à 107 µmol/l), un tableau de syndrome de détresse respiratoire aigue (rapport PaO2/FiO2 à 144) et une leucopénie à 2600 élts/mm^3^. Après un séjour de 10 Jours en réanimation son évolution a été favorable et le patient a été transféré au service de chirurgie générale pour complément de prise en charge.

## Discussion

Cette observation confirme que la péritonite secondaire est fréquemment rapportée dans la littérature. La perforation iléale par un corps étranger lors de la réduction d'une hernie inguinale peut être responsable d'une péritonite grave avec tableau de défaillance multiviscérale. Une péritonite est une inflammation ou une infection aiguë du péritoine. On parle de péritonite primitive lorsqu'il n'y a pas de lésion intra-abdominale responsable (exemple péritonite à pneumocoques, péritonite tuberculeuse). La péritonite est dite secondaire lorsqu'il existe un foyer intra-abdominal responsable de l'infection (perforation du tube digestif, ou d'un diverticule sigmoïdien, une infection de l'appendice ou de la vésicule biliaire...), elle peut aussi être consécutive à un acte opératoire abdominal [2, 3] ou à des corps étrangers intra-abdominaux [4]. Les causes des péritonites secondaires sont multiples. En faites c'est en général la perforation d'un viscère qui provoque la péritonite: perforation d'ulcère gastrique, perforation appendiculaire, perforation sigmoïdienne, perforation vésiculaire (lors d'une cholescystite négligée). Cette perforation peut être secondaire à une hernie étranglée [1]. Les signes fonctionnels sont les vomissements et l'arrêt des matières, éventuellement remplacés par une diarrhée fécale ou afécale. Les signes généraux sont ceux d'une infection qui devient rapidement grave. La péritonite se manifeste cliniquement par une réaction réflexe de la paroi abdominale. Cette manifestation réflexe est mise en évidence par la palpation de l'abdomen d'une défense ou d'une contracture. L'évolution peut être rapidement mortelle par état septique sévère, ou syndrome de défaillance polyviscérale. Il peut se produire des complications septiques locales avec formation d'abcès intra péritonéaux ou des abcès métastatiques hépatiques dus à une pyléphlébite.

Des complications septiques peuvent avoir lieu à distance: abcès métastatiques extra-abdominaux (rein, poumon, cerveau) et thrombophlébites périphériques. La péritonite est une urgence diagnostique et thérapeutique. Sa prise en charge thérapeutique comporte un volet médical et un volet chirurgical. Le traitement médical préopératoire et postopératoire comprend des antibiotiques et une réanimation pouvant s'associer à une alimentation parentérale. Le traitement chirurgical a plusieurs objectifs: supprimer la cause (exérèse appendiculaire, sigmoïdienne...) ou la neutraliser (drainage, extériorisation d'un segment digestif pour éviter de faire une anastomose digestive contre-indiquée dans ces conditions) et traiter la conséquence de l'infection par lavage péritonéal. Dans l'ensemble, le pronostic est plus grave si le malade est âgé, s'il est atteint de tares viscérales, en cas de péritonite stercorale et en cas de traitement chirurgical tardif [2, 3]. Notre cas est un peu particulier, en effet la cause la plus plausible de la perforation iléale serait un traumatisme par la cure dent lors de la réduction de l'hernie. En effet les constations per-opératoire oriente vers cette étiologie. L'exploration du grêle a permis de trouver un corps étranger (cure dent) sans perforation décelable. Cependant, l'épreuve aux bleu de méthylène a révélé une perforation grélique (iléale à 20 cm du carrefour iléo-coecal) et la chronologie de l'événement coïncident avec cette étiologie. Notre patient, bien qu'il ait développé un tableau de défaillance multiviscérale, son évolution a été favorable.

## Conclusion

La péritonite secondaire est fréquemment rapportée dans la littérature. Les causes sont multiples. La perforation iléale par un corps étranger lors de la réduction d'une hernie inguinale peut être responsable d'une péritonite grave avec tableau de défaillance multiviscérale.

## Conflits d’intérêts

Les auteurs ne déclarent aucun conflit d'itérêts.

## Contributions des auteurs

Tous les auteurs ont lu et approuvé la version finale du manuscrit.
